# Self-Reported Hearing Loss and Emotional Distress in Older Adults: Evidence from the Health and Retirement Study

**DOI:** 10.1093/geroni/igaf122.3411

**Published:** 2025-12-31

**Authors:** Hannah Crowder, Jessica West

**Affiliations:** Duke University School of Medicine, Durham, North Carolina, United States; Duke University School of Medicine, Durham, North Carolina, United States

## Abstract

Hearing loss (HL) is the most prevalent sensory disability in U.S. older adults and is associated with negative markers of emotional distress including depression, loneliness and anxiety. While research typically focuses on these global emotional states, less studied are emotions like anger. Anger tends to be experienced by older adults when they face changes in health status (e.g., HL). Understanding the extent to which HL is linked to anger expression is important, as translational studies have shown anger to be a predictor of negative health outcomes such as cardiovascular disease events and stroke. The current study uses wave-based mixed models to estimate 1) the association between HL and hearing aid use on expression of internalized anger (anger-in) and externalized anger (anger-out) and 2) how this association varies by gender using nationally-representative, longitudinal data from 10,721 adults aged 50 and older in the Health and Retirement Study (HRS) (2006-2012). The sample is predominantly female (68.4%) and participants self-reported normal hearing (85.1%), well-aided HL (1.3%), unaided HL (13.0%), or poorly aided HL (0.6%). Mixed effects models indicate that participants with unaided or poorly-aided HL reported greater anger-in (β=.06, p<.0001; β=.13, p<.010, respectively) and anger-out (β=.05, p<.001; β =.12, p≤.001, respectively) compared to those with normal hearing (no difference observed for well-aided HL), net of covariates. Greater on average anger-in (β=.03, p=.031) and anger-out (β=.05, p<.001) was experienced among male compared to female respondents. Findings underscore the importance of effective hearing interventions, as poor hearing—especially when untreated—is associated with increased anger expression.

